# Evaluating the Conversation Starter Kit in Long Term Care: A Canadian Perspective

**DOI:** 10.1093/geroni/igab046.3388

**Published:** 2021-12-17

**Authors:** Sharon Kaasalainen, Tamara Sussman

**Affiliations:** 1 McMaster University, Hamilton, Ontario, Canada; 2 McGill university, McGill University, Quebec, Canada

## Abstract

This study evaluated an advance care planning intervention, the Conversation Starter Kit (CSK) booklet, for use in long term care (LTC) homes. This study used a quasi-experimental, one group pre/post design. Quantitative surveys were administered before and after a 3-month advance care planning intervention (CSK booklet). Data were collected at three LTC homes in southern Ontario. We collected data from 55 resident who were able to make decisions on their own paired with 11 family members of these residents. We also collected data from 24 family members of residents who were not able to make decisions on their own. Quantitative surveys were administered before and after the intervention. An additional structured interview was completed at the end of the intervention period, which included both closed and open-ended questions to assess perceptions about the CSK booklet’s use or non-use. Residents reported higher engagement in advance care planning after having completed the CSK booklet than before, particularly related to asking questions to health care providers about health care decisions. Family members reported feeling very certain that they would be able to make decisions on behalf of the resident but they felt less certain after completing the CSK booklet, implying that the CSK booklet raised their awareness of the types of decisions that they might need to make, hopefully triggering them to become more prepared for these decisions in the future. The CSK appears acceptable, easy to use for residents and family members/friends in LTC, and can improve resident engagement in ACP.

